# Patientensteuerung und -autonomie: Ein ethisches Spannungsfeld?

**DOI:** 10.1007/s00103-026-04300-z

**Published:** 2026-09-21

**Authors:** Aline Weis, Andreas Bruns, Julia Sanchez, Eva C. Winkler

**Affiliations:** 1 Institut für Medizin- und Datenethik, Universität Heidelberg, Medizinische Fakultät, Tiergartenstraße 15, 69121 Heidelberg, Deutschland; 2 Nationales Centrum für Tumorerkrankungen (NCT), NCT Heidelberg, eine Partnerschaft zwischen DKFZ und dem Universitätsklinikum Heidelberg, Deutschland, Deutsches Krebsforschungszentrum (DKFZ) Heidelberg, Abteilung Gerechtigkeit und Gleichheit im Gesundheitswesen, Heidelberg, Deutschland; 3 Nuffield Council on Bioethics, London, Großbritannien

**Keywords:** Patientensteuerung, Patientenautonomie, Primärversorgung, Medizinethik, Freie Arztwahl, Patient steering, Patient autonomy, Medical ethics, Primary care, Free choice

## Abstract

Im Kontext aktueller gesundheitspolitischer Reformdebatten wird im deutschen Gesundheitswesen verstärkt über Instrumente der Patientensteuerung diskutiert. Diese zielen darauf ab, Zugangswege zur Versorgung zu koordinieren, Fehl- und Überversorgung zu reduzieren und Patientinnen und Patienten bedarfsgerecht durch ein komplexes Versorgungssystem zu leiten. Kritikerinnen und Kritiker sehen darin allerdings eine Einschränkung von Autonomie und freier Arztwahl.

Dieser Beitrag untersucht, ob und, wenn ja, inwiefern ein Konflikt zwischen Patientensteuerung und Autonomie besteht. Dabei wird ein Autonomieverständnis zugrunde gelegt, das nicht nur die Abwesenheit von Zwang oder Einschränkungen der Entscheidungsfreiheit umfasst, sondern auch die Befähigung zu wohlüberlegten Entscheidungen durch angemessene Information und Unterstützung einschließt.

Es wird gezeigt, dass Steuerungsmechanismen nicht zwangsläufig im Widerspruch zur Autonomie stehen. Sie können vielmehr Entscheidungsräume strukturieren, Orientierung verbessern und damit autonome Entscheidungen unterstützen. Empirische Befunde zeigen, dass formale Wahlfreiheit allein nicht garantiert, dass Patientinnen und Patienten Versorgungsangebote tatsächlich informiert und selbstbestimmt nutzen. Dagegen werden strukturierende Instrumente häufig als hilfreich wahrgenommen.

Für die ethische Bewertung ergeben sich drei zentrale Kriterien. Erstens ist zu prüfen, ob Steuerungsmechanismen präferenz- und wertbasierte Entscheidungen der Patientinnen und Patienten ermöglichen oder faktisch verunmöglichen. Zweitens ist ihr Beitrag zur Versorgungsqualität maßgeblich: Ethisch vertretbar ist Steuerung dann, wenn sie evidenzbasierte und koordinierte Versorgung fördert, ohne die Patientenversorgung zu verschlechtern. Drittens ist ihre Bedeutung für Gerechtigkeit und faire Ressourcenverteilung zu berücksichtigen. In einem solidarisch finanzierten Gesundheitssystem kann Patientensteuerung legitim sein, wenn sie dazu dient, begrenzte Ressourcen transparent, bedarfsgerecht und nachvollziehbar einzusetzen. Sind diese Kriterien erfüllt, gerät Patientensteuerung nicht in Konflikt mit dem Prinzip des Respekts vor der Patientenautonomie, sondern fördert diese im besten Fall.

## Abstract (en)

In the context of current debates in health policy, patient management is increasingly being discussed in the German healthcare system. These measures aim to coordinate access to care, reduce both inappropriate and over-provision of care, and guide patients through a complex healthcare system according to their needs. Critics, however, see this as a violation of patient autonomy and free choice.

This article examines whether, and if so, to what extent, there is a conflict between patient management and autonomy. The starting point is an analysis of whether and to what extent patient management conflicts with the principle of autonomy. This analysis is based on an understanding of autonomy that encompasses not only the absence of coercion or restrictions on freedom of choice, but also the ability to make well-considered decisions through appropriate information and support.

The article demonstrates that management mechanisms are not necessarily at odds with autonomy. Rather, they can structure decision-making spaces, improve orientation, and thus support autonomous decision-making. This is supported by empirical findings that show that formal freedom of choice alone does not guarantee that patients access care in an informed and self-determined way. Instead, structuring instruments are frequently perceived as helpful support.

The article then formulates three key criteria for the ethical evaluation of patient management. First, it must be examined whether management mechanisms enable or effectively prevent patients from making preference- and value-based decisions. Second, their contribution to the quality of care is crucial: control is ethically justifiable if it promotes evidence-based and coordinated care without worsening patient outcomes. Third, its significance for justice and fair resource allocation must be considered. In a solidarity-based healthcare system, patient management can be legitimate if it serves to allocate limited resources in a transparent, needs-based, and accountable manner. If these criteria are met, patient management does not conflict with the principle of respect for patient autonomy; rather, in the best-case scenario, it promotes it.

## Einleitung

Im Rahmen aktueller gesundheitspolitischer Reformdebatten werden verstärkt Maßnahmen diskutiert, die unter dem Begriff der „Patientensteuerung“ zusammengefasst werden können. Ziel solcher Maßnahmen ist es, die Inanspruchnahme medizinischer Leistungen besser zu koordinieren, Versorgungswege effizienter zu gestalten, Über‑, Unter- und Fehlversorgung zu reduzieren und eine bedarfsgerechte Nutzung begrenzter Ressourcen zu fördern. Ein Beispiel hierfür ist das Primärarztsystem, in dem die hausärztliche Praxis als erste Anlaufstelle dient und den Zugang zu weiterführender fachärztlicher Versorgung strukturiert [1]. Dadurch sollen unnötige Arztkontakte vermieden, Wartezeiten auf fachärztliche Termine verkürzt und Patientinnen und Patienten eine bessere Orientierung im zunehmend komplexen Gesundheitssystem ermöglicht werden [2]. Kontrovers diskutiert wird allerdings die Frage, ob und wie Maßnahmen der Patientensteuerung, wie das Primärarztsystem, mit der Autonomie oder Selbstbestimmung von Patienten und Patientinnen vereinbar sind. Denn auf den ersten Blick schränkt Steuerung die Wahlmöglichkeiten ein und damit unter Umständen auch die Freiheit, selbstbestimmt zu entscheiden. Der vorliegende Beitrag widmet sich dieser Frage, indem er den vermeintlichen Konflikt zwischen Steuerung und Autonomie näher untersucht. In Abschn. 2 wird zunächst der Autonomiebegriff erläutert, anschließend werden die begrifflichen Grundlagen und Voraussetzungen eines solchen Konflikts offengelegt. In Abschn. 3 werden Instrumente, Formen und strukturelle Rahmenbedingungen der Patientensteuerung in Deutschland anhand des Primärarztsystems exemplarisch aufgezeigt und empirische Befunde zur Sichtweise von Patienten und Patientinnen diskutiert. In Abschn. 4 werden drei mögliche Kriterien für eine ethische Bewertung des Konflikts zwischen Patientensteuerung und Autonomie formuliert. Der Artikel schließt mit der These, dass Patientensteuerung unter bestimmten Voraussetzungen nicht nur mit dem Prinzip des Respekts vor der Patientenautonomie vereinbar ist, sondern unter Umständen auch zu deren Förderung beitragen kann.

## Patientensteuerung – zwischen Autonomie und Paternalismus?

Das Prinzip der Autonomie schützt das moralische Recht der Patientinnen und Patienten, Entscheidungen über ihre Behandlung informiert und ohne Zwang selbst zu treffen. Auf den ersten Blick scheint Steuerung in direktem Konflikt mit diesem Prinzip zu stehen, insofern, als dass sie die Entscheidungsfreiheit von Patientinnen und Patienten einschränken kann.

Kritische Stimmen sehen in der Einführung eines Primärarztsystems beispielsweise eine Einschränkung der seit Jahrzehnten im deutschen Sozialrecht verankerten freien Arztwahl. Diese ist für gesetzlich Versicherte in § 76 Abs. 1 Sozialgesetzbuch  V normiert [3]. Dort wird festgelegt, dass Versicherte frei unter den zur vertragsärztlichen Versorgung zugelassenen Ärztinnen und Ärzten wählen können. Die gesetzliche Krankenversicherung ist damit traditionell durch ein hohes Maß an Wahlfreiheit gekennzeichnet [4], das mit der Einführung eines Primärarztsystems eingeschränkt werden würde. Ob eine solche Einschränkung allerdings tatsächlich die Autonomie von Patientinnen und Patienten gefährdet, hängt zunächst von dem zugrunde gelegten Autonomieverständnis ab.

### Was ist mit Patientenautonomie gemeint?

In der Medizinethik wird unter Autonomie in der Regel das Vermögen oder das moralische Recht verstanden, über Angelegenheiten, die das eigene Leben und die eigene Behandlung betreffen, selbst zu bestimmen. Als Vater des neuzeitlichen Autonomiebegriffs gilt Immanuel Kant, der den Begriff aus der politischen Philosophie entlehnt, wo er sich auf die Souveränität von Staaten bezieht, und als Vermögen von Personen zur vernünftigen Selbstgesetzgebung umdeutet [5]. Der kantische Autonomiebegriff wurde jedoch wegen seiner begrenzten Anwendbarkeit im klinischen Kontext kritisiert, nicht zuletzt, weil er hohe qualitative Anforderungen an autonome Entscheidungen stellen würde [6].

Andererseits sollte Autonomie auch nicht als rein formale Entscheidungsfreiheit verstanden werden, dass jede Entscheidung, unabhängig von ihrem Inhalt, als Ausdruck der Autonomie einer Person zu verstehen und zu respektieren sei [5]. Ein solches Autonomieverständnis trägt weder seinen kantischen Wurzeln Rechnung noch ist es auf die Praxis medizinischer Versorgung anwendbar, in der Entscheidungen in der Regel an bestimmte inhaltliche und rationale Anforderungen geknüpft sind. Zum Beispiel müssen autonome Entscheidungen ein Mindestmaß an Informiertheit, Nachvollziehbarkeit und Übereinstimmung mit den persönlichen Wertvorstellungen der Patientin oder des Patienten aufweisen, um als solche erkannt und respektiert werden zu können. In der Regel fordert das Autonomieprinzip von Ärztinnen und Ärzten nicht, die bloße Entscheidung einer Patientin oder eines Patienten zu respektieren, sondern durch Aufklärung und Gespräche sicherzustellen, dass die Entscheidung tatsächlich dem aufgeklärten Willen der Person entspricht. Dies gilt insbesondere, aber nicht ausschließlich bei weitreichenden Entscheidungen, etwa im Kontext lebensverlängernder Maßnahmen.

### Zur Abgrenzung von Paternalismus und Steuerung

Im klinischen Alltag werden diese qualitativen Anforderungen an autonome Entscheidungen nicht fortlaufend streng überprüft. Bei einwilligungsfähigen Erwachsenen besteht vielmehr grundsätzlich die Vermutung, dass ihre Entscheidungen als autonom zu respektieren sind. Dementsprechend hoch ist die Schwelle für die Rechtfertigung paternalistischer Interventionen, das heißt Einschränkungen der Entscheidungsfreiheit von Patientinnen und Patienten zu ihrem eigenen Wohl, aber gegen oder ohne ihren Willen [7]. Hier zeigt sich allerdings ein entscheidender Unterschied zwischen Paternalismus und Steuerung und zwischen den Logiken ihrer Rechtfertigung. Beim Paternalismus wird die Entscheidungsfreiheit der betreffenden Patientin bzw. des betreffenden Patienten unmittelbar hinsichtlich ihrer oder seiner eigenen Behandlung eingeschränkt. Dagegen betreffen Steuerungsmechanismen primär die Struktur der Zugangswege und Versorgungspfade. Sie gestalten die Rahmenbedingungen medizinischer Entscheidungen und wirken damit strukturell auf die Versorgung aller Patientinnen und Patienten. Individuelle Entscheidungen über die eigene Behandlung werden dadurch nicht aufgehoben, sondern innerhalb vorstrukturierter und evidenzbasierter Entscheidungsräume getroffen. Autonomie realisiert sich in diesem Sinne insbesondere in der Wahl zwischen medizinisch vertretbaren Optionen sowie in Entscheidungen über Therapieziele und Behandlungsbegrenzungen.

### Steuerung im Dienste der Versorgungsqualität und als autonomieförderndes Element

Die Annahme eines grundsätzlichen Konflikts zwischen Patientensteuerung und Autonomie beruht daher zumindest teilweise auf einer Verkürzung des Autonomiebegriffs. Dabei wird häufig unterstellt, dass jede strukturelle Vorgabe eine Einschränkung der Entscheidungsfreiheit der Patientinnen und Patienten darstelle. Tatsächlich ist die Entscheidungsfreiheit von Patientinnen und Patienten allerdings auch außerhalb von Steuerungsmechanismen vielfach strukturell eingeschränkt, ohne dass dies als autonomiegefährdend gilt. Patientinnen und Patienten können beispielsweise nicht frei über Medikamentendosierungen bestimmen oder verlangen, von ausschließlich männlichem bzw. weiblichem Personal behandelt oder über die Identität von Blutspenderinnen oder -spendern aufgeklärt zu werden. Solche Einschränkungen sind notwendige Strukturbedingungen verantwortlicher medizinischer Praxis und gelten als selbstverständlich mit dem Prinzip der Autonomie vereinbar.

Wie oben gezeigt, unterscheiden sich die Rechtfertigungslogiken von Paternalismus und Steuerung hier wesentlich. Während paternalistische Interventionen sich nur in eng begrenzten Situationen mit Rekurs auf das Wohl der individuellen Patientin bzw. des individuellen Patienten rechtfertigen lassen, stützt sich die Rechtfertigung von Steuerung auf systemische oder strukturelle Werte. Steuerung wird insbesondere unter Gesichtspunkten der Versorgungsqualität und Gerechtigkeit diskutiert [8]. Zum einen können Steuerungsmechanismen dazu beitragen, Patientinnen und Patienten schneller an die richtige Stelle im Gesundheitssystem zu bringen, unnötige Arztbesuche und Fehlversorgung zu reduzieren, und damit helfen, die Versorgungskapazitäten des Gesundheitssystems besser zu nutzen. Sie können dabei auch autonomiefördernd wirken, wenn sie Orientierung bieten und Entscheidungsprozesse strukturieren, ohne medizinisch vertretbare Optionen unangemessen einzuschränken oder individuelle Präferenzen und Wertvorstellungen unberücksichtigt zu lassen. Zum anderen stehen Gesundheitssysteme vor einer Vielzahl von Herausforderungen wie Fachkräftemangel, demografischem Wandel und steigendem Krankheitsaufkommen. Steuerung kann dabei helfen, knappe Ressourcen und Kapazitäten fairer zu verteilen und einen bedarfsorientierten und transparenten Zugang zur Versorgung herzustellen.

Autonomie realisiert sich also nicht als unbegrenzte Wahlfreiheit, sondern als die Möglichkeit, innerhalb evidenzbasierter und strukturierter Entscheidungsräume informierte und wertbasierte Entscheidungen zu treffen. Sie umfasst insbesondere die Wahl zwischen medizinisch gleichwertigen Behandlungsoptionen, die Festlegung von Therapiezielen sowie Entscheidungen über Beginn, Fortführung oder Begrenzung von Behandlungen. Ein Konflikt mit der Autonomie entsteht daher nicht durch die Strukturierung von Behandlungsprozessen, sondern erst dort, wo Entscheidungsräume unangemessen verengt werden oder diese keine ausreichende Berücksichtigung individueller Präferenzen und Wertvorstellungen mehr zulassen. Ob eine bestimmte Maßnahme der Steuerung autonomiegefährdend ist, muss also mit Blick auf diese Kriterien und unter Berücksichtigung der verfügbaren empirischen Evidenz diskutiert und entschieden werden.

## Zwischen Orientierung und Entscheidungsfreiheit: Empirische Befunde zur Patientensteuerung

### Instrumente und Formen der Patientensteuerung

Im deutschen Gesundheitssystem haben sich über die Zeit verschiedene Steuerungsmechanismen etabliert, die die Versorgungspfade von Patientinnen und Patienten strukturieren oder in ihrer Ausgestaltung mitbestimmen. Einige dieser Mechanismen wurden bereits flächendeckend implementiert, während andere nur regional und/oder als Pilotprojekte umgesetzt wurden. Das in der Einleitung erwähnte Primärarztmodell stellt hierbei nur eine Ausprägung dar. Tab. 1 bietet einen Überblick über weitere Instrumente der Patientensteuerung, die nach den Phasen des Versorgungsprozesses geordnet sind, in denen sie jeweils wirksam werden. Die Darstellung erhebt keinen Anspruch auf Vollständigkeit, sondern versteht sich als systematische Annäherung, um die Vielfalt existierender Steuerungsformen sowie deren Einbettung in unterschiedliche Phasen des Versorgungspfades zu verdeutlichen.

**Tab. 1:** Steuerungsinstrumente nach Phasen im Versorgungspfad

Versorgungsphase	Steuerungsinstrument
Zugang zur Versorgung	Hausarztzentrierte Versorgung/Primärarztmodell
	Terminservicestellen/Überweisungspflichten
	KV-Nummer 116117
	Zuzahlungen
	Steuerung in der Notfallversorgung
	Gesundheitskioske
Indikationsstellung/Bewilligung von Leistungen	Gutachterverfahren (Psychotherapie/Reha)
	Psychotherapeutische Sprechstunde
Koordination und sektorenübergreifende Steuerung	Integrierte Versorgung/Selektivverträge Patientenlotsen
	Durchgangsarzt (Unfallversicherung)

Einige dieser Instrumente – etwa die hausarztzentrierte Versorgung (HzV), das Durchgangsarztverfahren, Gutachterverfahren im Bereich der Psychotherapie oder Rehabilitation, Regelungen der Notfallversorgung sowie Terminservicestellen – sind mit einem definierten Koordinations- oder Steuerungsauftrag verbunden. Andere Instrumente wie Zuzahlungen oder Gesundheitskioske haben primär eine andere Zielsetzung, können jedoch ebenfalls eine steuernde Wirkung auf Versorgungspfade entfalten. Die mit diesen Mechanismen verbundenen Zielsetzungen reichen von der Koordination der Versorgung über die Beeinflussung von Ressourcenallokation und Inanspruchnahmeverhalten sowie die Reduktion von Wartezeiten bis hin zur Unterstützung von Navigation und Orientierung in einem durch hohe Komplexität gekennzeichneten Gesundheitssystem.

### Strukturelle Rahmenbedingungen des deutschen Gesundheitssystems

Patientensteuerung im deutschen Gesundheitswesen und deren mögliche Folgen für die Patientenautonomie sind somit nur vor dem Hintergrund der spezifischen Systemstrukturen zu verstehen. Prägend sind dabei insbesondere der traditionell weitgehend freie Zugang zu ärztlicher Versorgung, die sektorale Trennung zwischen ambulanter und stationärer Versorgung sowie das Prinzip der Selbstverwaltung.

Das im Sozialrecht verankerte Recht auf freie Arztwahl existiert bereits seit 1955 und wird verfassungsrechtlich über die allgemeine Handlungsfreiheit nach Art. 2 Abs. 1 Grundgesetz abgesichert [6]. Diese weitreichende Wahlfreiheit ermöglicht Versicherten den unmittelbaren Zugang zu unterschiedlichen Versorgungsangeboten sowie einen freien Wechsel zwischen Ärztinnen und Ärzten. Sie kann jedoch zugleich zu unkoordinierten, mehrfachen oder medizinisch nicht erforderlichen Inanspruchnahmen führen und damit das Wirtschaftlichkeitsgebot als leitendes Prinzip des Krankenversicherungsrechts berühren. Dieses verlangt, dass Leistungen ausreichend, zweckmäßig und wirtschaftlich sind und das Maß des Notwendigen nicht überschreiten. Steuerungsinstrumente können vor diesem Hintergrund dazu dienen, die freie Arztwahl mit einer bedarfsgerechten, koordinierten und ressourcenschonenden Versorgung zu verbinden. Zugleich verlagert ein weitgehend offener Zugang erhebliche Anforderungen an Orientierung, Koordination und Entscheidungsfindung auf die Patientinnen und Patienten [9]. Das Prinzip der Selbstverwaltung begrenzt darüber hinaus direkte staatliche Steuerung und überträgt die Verantwortung für die konkrete Ausgestaltung der Versorgungsprozesse in erheblichem Maße auf die Akteure des Gesundheitssystems.

### Wahlfreiheit, Orientierung und navigationale Gesundheitskompetenz

Insgesamt schaffen diese strukturellen Merkmale ein System mit hoher formaler Wahlfreiheit, in dem Steuerungsanforderungen jedoch in erheblichem Maße auf Patientinnen und Patienten selbst verlagert sind. Daraus ergibt sich, dass die Komplexität des Systems dazu führen kann, dass die vermeintliche Freiheit des offenen Zugangs, die sich durch die freie Arztwahl ergibt, durchaus als eine Freiheit unter hohen Anforderungen an Informationsbeschaffungs- und Navigationskompetenz verstanden werden kann. Empirische Befunde zur sogenannten navigationalen Gesundheitskompetenz stützen diese Einschätzung. So zeigen Schaeffer et al., dass mehr als drei Viertel der Bevölkerung in Deutschland sich schlecht im Gesundheitssystem zurechtfinden [10]: „Bei 82 % der Bevölkerung fällt die Navigationale Gesundheitskompetenz negativ aus.“ Besonders ausgeprägt sei diese Problematik bei Personen mit niedrigem sozioökonomischen Status, finanziellen Belastungen oder geringem Bildungsniveau [11].

Auch Daten der Stiftung Unabhängige Patientenberatung Deutschland weisen auf erhebliche Orientierungs- und Zugangsprobleme hin [12]. Ein Großteil der Beratungsanfragen zu Leistungserbringern betrifft die Suche insbesondere nach Facharztpraxen, Therapieplätzen oder einer kontinuierlichen hausärztlichen Versorgung. In den entsprechenden Beratungsgesprächen berichten Ratsuchende wiederholt von Schwierigkeiten, passende und qualifizierte Angebote zu identifizieren sowie zeitnah Termine zu erhalten, was auf strukturelle Hürden beim Zugang zur Versorgung hindeutet. Vor diesem Hintergrund erscheint die Annahme, dass Patientinnen und Patienten die bestehende Wahlfreiheit in jedem Fall im Sinne autonomer, informierter Entscheidungen nutzen können, empirisch nur eingeschränkt haltbar.

Ein Beispiel dafür, wie Patientinnen und Patienten die Struktur ihres Versorgungszugangs selbst mitbestimmen können, bietet Dänemark. Dort wählen Versicherte zwischen einem Modell mit Bindung an eine Hausarztpraxis und hausärztlich gesteuertem Zugang zur spezialisierten Versorgung sowie einem Modell mit weitgehend freiem Zugang zu Hausärztinnen und Hausärzten sowie Facharztpraxen, das jedoch mit Eigenbeteiligungen verbunden ist [13]. Dass mehr als 99 % der Bevölkerung dem ersten Modell angehören, unterstreicht dessen prägende Stellung im dänischen Versorgungssystem, erlaubt jedoch keine Rückschlüsse auf die individuellen Motive dieser Entscheidung. Ein ähnliches Bild zeigt eine repräsentative Befragung in Deutschland, wonach ein erheblicher Anteil der Bevölkerung bereit wäre, Einschränkungen der freien Arztwahl zugunsten eines schnelleren Zugangs zur Versorgung in Kauf zu nehmen [14]. Auch empirische Befunde von Schüttig et al. stützen diese Differenzierung. So zeigen Ergebnisse der PFAD-Studie, dass Patientinnen und Patienten koordinierte und kontinuierliche Versorgung häufig höher gewichten als maximale Wahlfreiheit bei der Inanspruchnahme von Leistungserbringenden [15]. Zugleich besteht ein ausgeprägtes Bedürfnis nach Orientierung und Mitentscheidung bei strukturierten Versorgungsangeboten.

### Wahrnehmung und Akzeptanz von Steuerungsmechanismen

Von Bedeutung ist zudem die Frage, inwieweit Patientinnen und Patienten sich der jeweiligen Steuerungsmechanismen bewusst sind und deren Wirkung auf ihr Inanspruchnahmeverhalten nachvollziehen können. Ein wesentlicher Teil der Befragten innerhalb der oben stehend erwähnten PFAD-Studie beispielsweise war sich über die eigene Teilnahme an den untersuchten strukturierten Versorgungsmodellen nicht im Klaren oder äußerte weiteren Beratungsbedarf [15]. Dies weist auf generelle Informations- und Transparenzdefizite hin, die auch hinsichtlich der mit den Versorgungsmodellen verbundenen Steuerungsmechanismen bestehen können.

Gleichzeitig zeigen Analysen von Abrechnungsdaten, dass Unterschiede bei der Inanspruchnahme ambulanter Leistungen in Deutschland überwiegend auf Faktoren wie Gesundheitszustand und Präferenzen der Patientinnen und Patienten zurückzuführen sind [16]. Dies deutet darauf hin, dass Patientinnen und Patienten ihr Inanspruchnahmeverhalten grundsätzlich aktiv gestalten, wenngleich bei komplexeren Versorgungssituationen auch angebotsseitige Faktoren eine relevante Rolle spielen.

Auch die verbreitete Annahme, dass freie Arztwahl unmittelbar mit einem höheren Vertrauen in die behandelnde Ärztin oder den behandelnden Arzt einhergehe, lässt sich empirisch bislang nur begrenzt stützen. Eine systematische Übersichtsarbeit aus dem Cochrane-Kontext zeigt, dass derzeit keine ausreichende Evidenz vorliegt, um den Einfluss spezifischer Interventionen auf das Vertrauen von Patientinnen und Patienten verlässlich zu bestimmen [17]. Dabei wird ausdrücklich darauf hingewiesen, dass die vorhandene Studienlage durch methodische Heterogenität, unterschiedliche Messinstrumente sowie ein insgesamt hohes Vertrauensniveau in der Bevölkerung begrenzt ist. Ein direkter Zusammenhang zwischen Wahlmöglichkeiten im Versorgungssystem und Vertrauen wird in diesem Kontext bislang nicht belastbar belegt. Dies legt nahe, dass Vertrauen weniger von der formalen Ausgestaltung von Wahloptionen als vielmehr von Faktoren wie Kommunikation, Kontinuität der Versorgung und wahrgenommener Behandlungsqualität abhängen könnte.

### Wirkungen strukturierender Versorgungsangebote und Grenzen der Evidenz

Hinweise darauf, dass strukturierende Elemente der Patientensteuerung nicht zwangsläufig als Einschränkung, sondern auch als Unterstützung wahrgenommen werden können, liefern zudem Studien zu Navigations- und Koordinationsangeboten [18]. So berichten Patientinnen und Patienten in Evaluationsstudien entsprechender Programme von einer hohen Zufriedenheit, insbesondere wenn diese auf Koordinationserfordernisse und soziale Unterstützungsbedarfe ausgerichtet sind und damit zur Bewältigung komplexer Versorgungssituationen beitragen.

Analysen aus einzelnen Versorgungsbereichen, etwa der Onkologie, zeigen, dass die Inanspruchnahme medizinischer Leistungen bereits heute vielfach durch die jeweilige Indikation und den Behandlungsverlauf strukturiert wird [19]. Diese faktische Steuerung beruht jedoch nicht durchgehend auf explizit formulierten, sektorenübergreifenden Versorgungspfaden. Insbesondere Leitlinien enthalten bislang nur wenige konkrete Vorgaben dazu, wie Patientinnen und Patienten zwischen ambulanter, stationärer und spezialisierter Versorgung koordiniert werden sollen. Für die Entwicklung weitergehender Steuerungsinstrumente bleibt daher zu klären, anhand welcher medizinischen, organisatorischen und patientenbezogenen Kriterien Versorgungswege gestaltet werden sollten.

Insgesamt legen diese Befunde nahe, dass Patientensteuerung nicht als Einschränkung von Selbstbestimmung zu verstehen ist, sondern auch als strukturierendes Element, das dazu beitragen kann, Orientierung zu verbessern und damit die Voraussetzungen für informierte, präferenz- und wertbasierte Entscheidungen überhaupt erst zu schaffen. Es ist jedoch auch festzuhalten, dass die internationale empirische Evidenz zu den Wirkungen von Steuerungsmechanismen wie Gatekeeping uneinheitlich und nur begrenzt belastbar ist. Während einzelne Studien Hinweise auf Effekte etwa hinsichtlich Inanspruchnahme, Kosten und Versorgungsqualität liefern, bleibt die Evidenz zu patientenbezogenen Endpunkten wie Zufriedenheit oder gesundheitliche Outcomes bislang uneindeutig und die zugrunde liegende Datenlage wird in systematischen Übersichtsarbeiten weiterhin als heterogen und methodisch eingeschränkt beschrieben [20, 21].

## Zwischen Autonomie und Steuerung: Kriterien einer ethischen Bewertung

Die vorangegangenen Ausführungen legen nahe, dass die Frage nach einem Spannungsverhältnis zwischen Patientensteuerung und Autonomie nicht pauschal zu beantworten ist, sondern einer differenzierten normativen Einordnung bedarf. Hierfür lassen sich mehrere zentrale Bewertungsdimensionen heranziehen.

Erstens ist zu prüfen, inwieweit Steuerungsmechanismen die Autonomie von Patientinnen und Patienten unter strukturellen Bedingungen wahren. Maßgeblich ist dabei, ob sie präferenz- und wertbasierte Entscheidungen ermöglichen oder diese faktisch verunmöglichen. Zweitens ist der Beitrag zur Versorgungsqualität zu berücksichtigen. Steuerung kann dann als ethisch gerechtfertigt gelten, wenn sie evidenzbasierte, bedarfsorientierte und koordinierte Versorgung unterstützt und zu einer Verbesserung von Versorgungsprozessen beiträgt, ohne dabei die Versorgungsqualität zu verschlechtern. Drittens ist die Frage der Gerechtigkeit und der fairen Ressourcenverteilung zentral. Insbesondere in einem solidarisch finanzierten Gesundheitssystem können Steuerungsmechanismen eine legitime Funktion erfüllen, wenn sie dazu beitragen, begrenzte Ressourcen transparent, bedarfsgerecht und nachvollziehbar einzusetzen.

Ergänzend sind Kriterien wie Transparenz, Nachvollziehbarkeit und die Möglichkeit zur Beteiligung von Patientinnen und Patienten an der Ausgestaltung von Steuerungsmechanismen zu berücksichtigen, da sie die Akzeptanz und Legitimität entsprechender Eingriffe maßgeblich beeinflussen. Insgesamt wird deutlich, dass Patientensteuerung nicht per se im Widerspruch zur Autonomie steht, sondern unter bestimmten Bedingungen auch als deren strukturelle Voraussetzung verstanden werden kann.

## Fazit

Die vorliegenden Überlegungen sprechen gegen die Annahme eines notwendigen oder prinzipiellen Konflikts zwischen Patientensteuerung und Patientenautonomie. Ein solcher ergibt sich vor allem dann, wenn Autonomie verkürzt als bloße Abwesenheit von Einschränkungen der Wahlfreiheit verstanden wird. Ein differenzierteres Autonomieverständnis, das auch die Voraussetzungen informierter und wohlüberlegter Entscheidungen einbezieht, eröffnet hingegen die Möglichkeit, Steuerungsmechanismen nicht nur als potenzielle Einschränkung, sondern auch als legitimes Instrument zu begreifen. Patientensteuerung ist dann ethisch gerechtfertigt, wenn sie die Ausübung präferenz- und wertbasierter Entscheidungen nicht aufhebt, sondern in strukturierter Weise unterstützt, zur Versorgungsqualität beiträgt und begrenzte Ressourcen transparent und bedarfsgerecht einsetzt.

Mit Blick auf konkrete Ausgestaltungen zeigt sich zudem, dass Einschränkungen der Wahlfreiheit nicht zwingend als Verlust von Selbstbestimmung wahrgenommen werden. Vielmehr können strukturierende Elemente, etwa im Rahmen eines Primärarztsystems, von Patientinnen und Patienten auch als Orientierungshilfe gesehen werden, die den Umgang mit komplexen Versorgungssituationen erleichtert und Entscheidungsprozesse unterstützt.

Die bislang uneinheitliche empirische Evidenz erlaubt jedoch keine pauschale Bewertung; die Wirkungen konkreter Steuerungsinstrumente müssen daher jeweils kontextbezogen geprüft werden. Damit verschiebt sich die Perspektive von der Frage, ob Steuerung Autonomie einschränkt, hin zu der Frage, unter welchen Bedingungen sie zu deren Realisierung beitragen kann.

Für die weitere Ausgestaltung von Steuerungsmechanismen ergibt sich daraus die Notwendigkeit, die betroffenen Patientengruppen stärker in entsprechenden Gestaltungsprozesse einzubeziehen. Eine solche partizipative Weiterentwicklung, etwa mit Formaten der Bürgerbeteiligung, kann dazu beitragen, die Legitimität, Akzeptanz und praktische Wirksamkeit der Patientensteuerung zu erhöhen und zugleich sicherzustellen, dass entstehende Strukturen tatsächlich zur Verbesserung der Versorgung und zur Eröffnung sinnvoller Entscheidungsräume beitragen.

## Data Availability

Für diesen Artikel wurden keine Datensätze erstellt oder analysiert.
